# Questionable necessity effects on creativity in the workplace: a comment on Mercier and Lubart (2026)

**DOI:** 10.3389/fpsyg.2026.1832547

**Published:** 2026-06-02

**Authors:** Kimmo Sorjonen, Bo Melin, Marika Melin

**Affiliations:** Department of Clinical Neuroscience, Karolinska Institutet, Stockholm, Sweden

**Keywords:** analytic rigor, creativity, necessary condition analysis (NCA), ranges of spuriousness, scrutiny of findings

## Necessary condition analysis (NCA)

**Figure 1:**
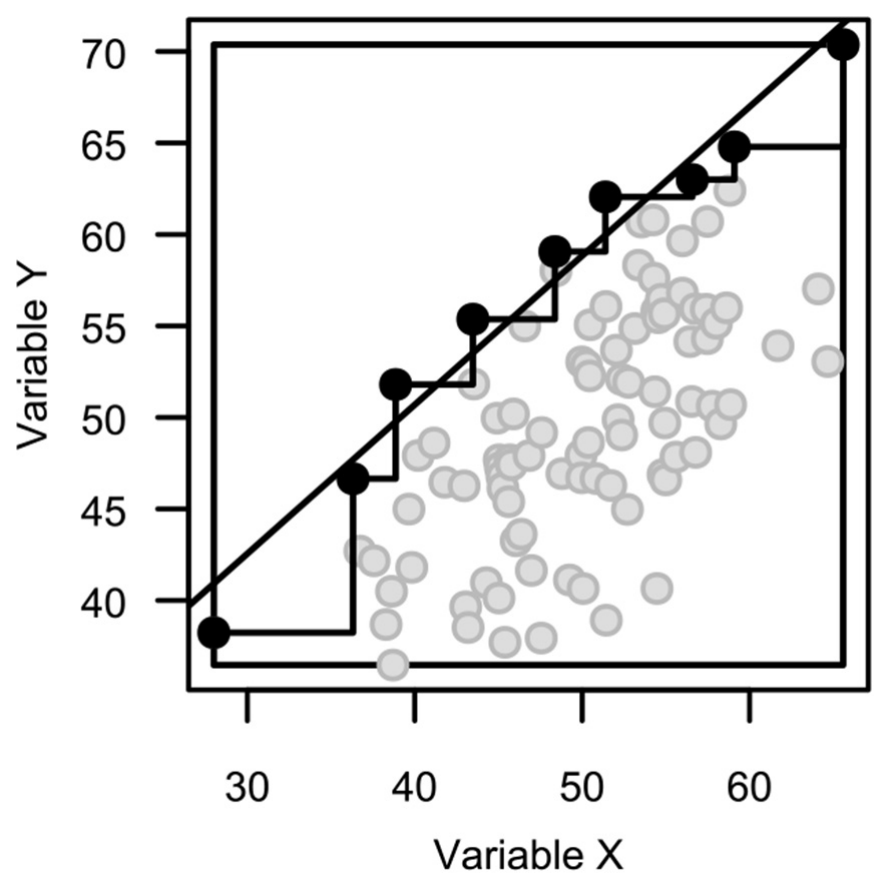
NCA identifies ceiling points with a higher *Y*-value than all observation points with a lower *X*-value (the solid markers). Necessity effects are estimated as the size of the (semi) empty space above the step function (CE-FDH) or the linear regression function (CR-FDH). Simulated data.

Necessary condition analysis (NCA) identifies so-called ceiling points, which are observation points with a higher *Y*-value than all observation points with a lower *X*-value (see solid markers in Figure 1). Then, either a step-function or a linear regression function is drawn through these ceiling points, thus separating a (semi) empty space in the upper-left corner of the *XY*-plot. A necessity effect is estimated by dividing the size of the (semi) empty space by (*X*_max_-*X*_min_)(*Y*_max_-*Y*_min_). If using the step function, the necessity effect is called ceiling envelopment with free disposal hull (CE-FDH) and if using the linear regression function it is called ceiling regression with free disposal hull (CR-FDH). The size of the (semi) empty space and the necessity effect is assumed to indicate to what degree a low value on *X* precludes a high value on *Y* (Dul, 2016). Statistical significance of the necessity effect can be estimated through permutation (Dul et al., 2020). For example, in Figure 1, CE-FDH = 619.1/1,274.6 = 0.49 (*p* < 0.001) and CR-FDH = 532.8/1,274.6 = 0.42 (*p* < 0.001).

Mercier and Lubart (2026) analyzed data on creativity in the workplace with NCA. Data was collected from *N* = 1,384 workers in France (88.1 % female, mean age = 40.2 years). Based on statistically significant necessity effects, Mercier and Lubart concluded that creativity self-efficacy and, to a lesser degree, creative process engagement, creative personal identity, openness to experience, and creative personality appeared necessary for creativity.

## Range of spuriousness

Analyses have established that necessity effects in NCA may be spurious and do, consequently, not prove necessity. An empty space in the upper-left corner of a XY-plot may be due to a correlation between *X* and *Y* which could, in turn, be due to confounding by a third variable *Z*. Necessity effects in NCA do, consequently, not require necessity and do not prove necessity any more than correlations prove causality (Sorjonen and Melin, 2025).

We have recommended users of NCA to scrutinize their findings by estimating “ranges of spuriousness” and to require that necessity effects are above this range before claiming necessity. With this methodology, a necessity effect (CE-FDH or CR-FDH) is estimated in a large number (e.g., 1,000) of generated datasets with the same sample size and correlation between *X* and *Y* as in the original dataset. The range of spuriousness corresponds to a confidence interval across these estimated necessity effects. If the original empirical necessity effect falls within or below this range, it is not significantly stronger than could be expected solely due to the correlation between the variables even without *X* being necessary for *Y*. If this happens, the null hypothesis of no necessity should be retained. A range of spuriousness may be likened to a control group. In order to claim a treatment effect, the treatment group should differ significantly from the control group rather than, which is the present norm in NCA, being content with a significant difference from zero (Sorjonen and Melin, 2025).

The permutation test in NCA evaluates if the necessity effect differs significantly from zero. This corresponds to evaluating, for example, if a group taking an allegedly enhancing drug can lift more than zero kilos on average. Our proposed method, with estimations of ranges of spuriousness, on the other hand, evaluates if the necessity effect differs significantly from an effect that can be expected solely due to the correlation between *X* and *Y*. This corresponds to comparing a group taking an allegedly enhancing drug with a control group not taking the drug rather than comparing with zero. As people can be assumed to lift more than zero kilos even without enhancing drugs, a comparison with a control group makes more sense than comparing with zero. Likewise, as necessity effects in the NCA increase in size with the correlation between *X* and *Y*, even without *X* being necessary for *Y* (Sorjonen and Melin, 2025), it makes more sense to compare with the range of spuriousness (i.e., the size of the necessity effect that can be expected due to the correlation between *X* and *Y*) than to compare with zero. Just like it is necessary to use a control group in order to evaluate the effectiveness of an allegedly enhancing drug, ranges of spuriousness should be estimated in order to identify genuine (i.e., non-spurious) necessity effects in the NCA.

By estimating ranges of spuriousness we have shown that claimed necessity effects of performance expectancy and social influence on acceptance of surgical robots (Andrés-Sánchez et al., 2025), of basic psychological need satisfaction on wellbeing at work (Ding and Kuvaas, 2025; Olafsen and Marescaux, 2025), and of perceived burdensomeness and thwarted belonging on suicidal ideation (Marchetti et al., 2026) may have been spurious (Sorjonen et al., 2026a,b, 2025a,b).

## Scrutiny of Mercier and Lubart (2026)

**Figure 2:**
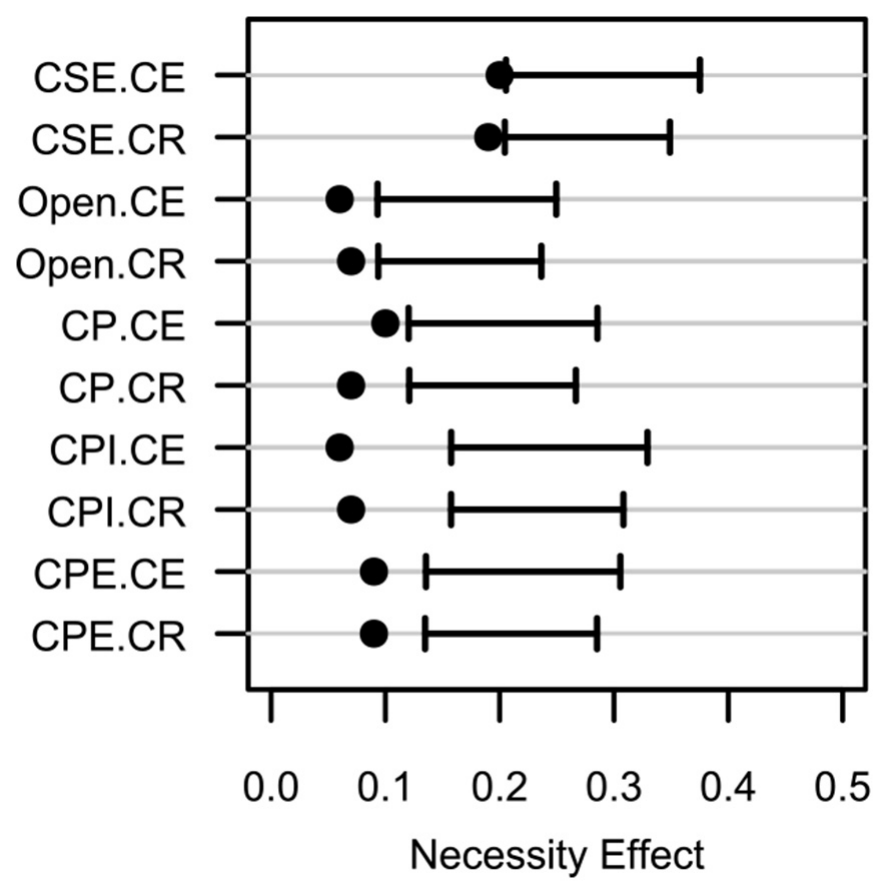
Necessity effects on creativity in the workplace reported by Mercier and Lubart (the solid markers) as well as ranges of spuriousness (i.e., necessity effects that can be expected solely due to correlations between the variables) estimated by us (the whiskers). CE/CR = CE-FDH and CR-FDH, respectively; CSE, creativity self-efficacy; Open, openness to experience (trait); CP, creative personality; CPI, creative personal identity; CPE, creative process engagement.

Following the methodology outlined above, we estimated ranges of spuriousness by estimating necessity effects in 1,000 generated datasets with the same sample size (*N* = 1,384) and correlations between variables as in the data used by Mercier and Lubart (2026). Analyses were conducted with R 4.4.3 statistical software (R Core Team, 2026) using the MASS (Venables and Ripley, 2002), NCA (Dul, 2026), and lavaan (Rosseel, 2012) packages. Our analytic script, which also generates the used data, is available at the Open Science Framework at https://osf.io/dhyef/. All necessity effects reported by Mercier and Lubart fell below the estimated ranges of spuriousness (Figure 2), meaning that they were significantly weaker than could be expected solely due to correlations between the variables. This corresponds to individuals in a treatment group experiencing a worse outcome than individuals in a control group.

## Alternative model

**Figure 3:**
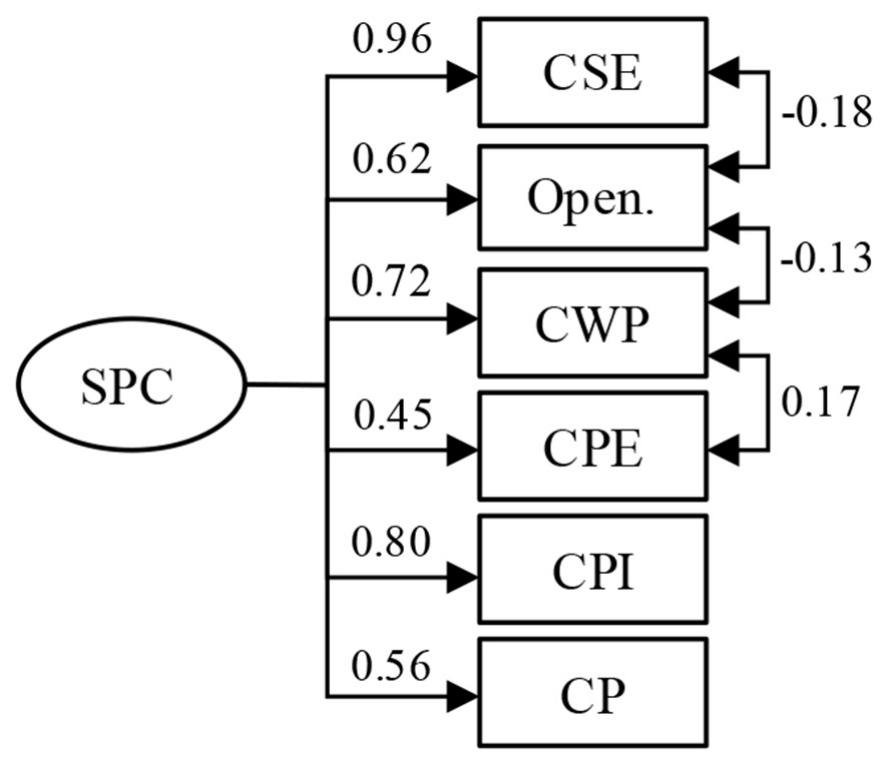
Alternative model where all measures are indicators of a latent variable. The model had excellent fit [χ^2^ = 13.0, DF = 12, *p* = 0.369, *CFI* = 1.000, *TLI* = 1.000, *RMSEA* = 0.008 (90% CI: 0.000; 0.029)]. Standardized estimates, all highly significant (*p* < 0.001). SPC, self-perceived creativity; CSE, creativity self-efficacy; Open, openness to experience (trait); CWP, creativity in the workplace; CPE, creative process engagement; CPI, creative personal identity; CP, creative personality.

Instead of assuming genuine (i.e., non-spurious) necessity effects on creativity in the workplace, a conclusion not supported by the findings reported above, we propose that the data analyzed by Mercier and Lubart, and reanalyzed by us, may have been generated by the model in Figure 3. Here, all measured scores are assumed to be indicators of a common self-perceived creativity latent variable. However, the model does not include any direct effects between the measures, meaning that such effects identified by statistical analyses (e.g., regression analyses or NCA) would be spurious. The model had excellent fit. It is important to note that data generation in line with Figure 3 would be sufficient to explain the necessity effects reported by Mercier and Lubart and that no additional conditions, e.g., true necessity effects between the measures, would need to be fulfilled.

## Summary and concluding remarks

We set out to scrutinize findings and claimed necessity effects on creativity in the workplace by Mercier and Lubart. We found that all of their reported necessity effects fell below ranges of spuriousness, meaning that they were significantly weaker than could be expected solely due to correlations between the variables. Hence, correlations could account for the necessity effects, the findings by Mercier and Lubart may have been spurious, and their conclusions premature.

The present findings carry methodological, theoretical, and also potentially practical relevance. Firstly, they show, together with our previous challenges (Sorjonen et al., 2026a,b, 2025a,b; Sorjonen and Melin, 2025), that necessity is not necessary for necessity effects in NCA. This is important for researchers using NCA to bear in mind in order not to overinterpret findings. Secondly, they indicate that creativity self-efficacy, creative process engagement, creative personal identity, openness to experience, creative personality, and creativity in the workplace may all be indicators of the same overarching self-perceived creativity latent variable, rather than being independent variables in a causal network. Thirdly, the present findings suggest that it may not be necessary for employers to search for people who give “the right” answers on various personality inventories, e.g., measuring creativity self-efficacy, in order to find creative employees. Lack of high self-perceived creativity might, possibly, be compensated by other creativity enhancing characteristics, e.g., being knowledgeable and dedicated.

## Limitations

The present findings do not prove, once and for all, the null hypothesis that creativity self-efficacy, creative process engagement, creative personal identity, openness to experience, and creative personality are not necessary for creativity in the workplace. The more limited conclusion to be drawn is that the data used by Mercier and Lubart does not support rejecting the null hypothesis.

In most situations, to prove necessity appears almost impossible, as it would require observing all data. For example, to prove that all swans are white (i.e., that being white is necessary for being a swan) would require observing all swans. Hence, we would not recommend researchers to claim necessity with a high degree of confidence even if their estimated necessity effects would fall above estimated ranges of spuriousness. Our method should, in our opinion, be used as a tool to challenge questionable necessity claims rather than to prove necessity.

## Conclusions

Here, we showed that necessity effects of creativity self-efficacy, creative process engagement, creative personal identity, openness to experience, and creative personality on creativity in the workplace could be accounted for by correlations between the variables. Hence, findings reported by Mercier and Lubart may have been spurious and their conclusions of necessity premature.

It is important for users of NCA to be aware that necessity is not required for necessity effects in NCA. A correlation between *X* and *Y*, which could, in turn, be due to confounding by a third variable *Z*, is sufficient for a necessity effect in NCA. Hence, necessity effects in NCA do not prove necessity any more than correlations prove causality. We recommend researchers using NCA to scrutinize their findings by estimating, as we did here, ranges of spuriousness and to require that necessity effects fall above this range before claiming necessity. Otherwise, the necessity effect is not significantly stronger than could be expected due to the correlation between the variables. This would correspond to a treatment group not differing significantly from a control group. The NCArigour function available in our analytic script at https://osf.io/dhyef/ can be used to estimate ranges of spuriousness and to scrutinize necessity effects.
